# Myotonic Dystrophy type 1 cells display impaired metabolism and mitochondrial dysfunction that are reversed by metformin

**DOI:** 10.18632/aging.103022

**Published:** 2020-04-08

**Authors:** Mikel García-Puga, Ander Saenz-Antoñanzas, Roberto Fernández-Torrón, Adolfo Lopez de Munain, Ander Matheu

**Affiliations:** 1 Neuroscience Area, Biodonostia Health Research Institute, San Sebastian, Spain; 2 Cellular Oncology Group, Biodonostia Health Research Institute, San Sebastian, Spain; 3 Neurology Department, Donostia University Hospital, OSAKIDETZA, San Sebastian, Spain; 4 IKERBASQUE, Basque Foundation for Science, Bilbao, Spain; 5 CIBERfes, Carlos III Institute, Madrid, Spain; 6 CIBERNED, Carlos III Institute, Madrid, Spain; 7 Faculty of Medicine and Nursery, Department of Neurosciences, University of the Basque Country, San Sebastian, Spain

**Keywords:** myotonic dystrophy type 1, aging, metabolism, metformin, mitochondria, neuromuscular disease

## Abstract

Myotonic dystrophy type 1 (DM1; MIM #160900) is an autosomal dominant disorder, clinically characterized by progressive muscular weakness and multisystem degeneration. The broad phenotypes observed in patients with DM1 resemble the appearance of a multisystem accelerated aging process. However, the molecular mechanisms underlying these phenotypes remain largely unknown. In this study, we characterized the impact of metabolism and mitochondria on fibroblasts and peripheral blood mononuclear cells (PBMCs) derived from patients with DM1 and healthy individuals. Our results revealed a decrease in oxidative phosphorylation system (OXPHOS) activity, oxygen consumption rate (OCR), ATP production, energy metabolism, and mitochondrial dynamics in DM1 fibroblasts, as well as increased accumulation of reactive oxygen species (ROS). PBMCs of DM1 patients also displayed reduced mitochondrial dynamics and energy metabolism. Moreover, treatment with metformin reversed the metabolic and mitochondrial defects as well as additional accelerated aging phenotypes, such as impaired proliferation, in DM1-derived fibroblasts. Our results identify impaired cell metabolism and mitochondrial dysfunction as important drivers of DM1 pathophysiology and, therefore, reveal the efficacy of metformin treatment in a pre-clinical setting.

## INTRODUCTION

Myotonic dystrophy is the most common type of muscular dystrophy in adults and is inherited in an autosomal dominant manner [1]. There are two clinically similar but genetically distinct types: DM type 1 (DM1, also known as Steinert’s disease; MIM #160900), caused by an unstable expansion of a CTG trinucleotide repeat in the noncoding region of the dystrophia myotonic-protein kinase gene (*DMPK*) [2], and DM type 2 (DM2; MIM #602668), caused by a tetra-nucleotide repeat CCTG expansion in the zinc finger 9 (*ZNF9*) gene [3]. CTG and CCTG expansions lead to formation of transcript aggregates in the nucleus, which interfere with proteins that play an important role in RNA metabolism, including members of the muscleblind (MBNL) and CUGBP RNA-Binding Protein Elav-Like Family Member 1 (CELF1) families of RNA-binding proteins [4]. Both diseases are characterized by missplicing of several downstream effector genes with negative effects on multiple tissues, thus contributing to the multisystem pathogenesis of DM [5]. DM1 is more common than DM2 and represents a more severe phenotype. In DM1, unaffected individuals carry less than 50 triplet repeats, whereas expansions ranging between 50 and 4000 CTG repeats have been found in affected individuals [6].

Patients with DM1 present a multisystem degenerative process that includes progressive muscular weakness and atrophy, myotonia, cardiomyopathy, insulin-resistance, cataracts, increased cancer incidence, neurodegeneration, metabolic syndrome, or premature death. This multisystem degenerative process strongly resembles an accelerated aging process [7, 8]. From a cellular point of view, different pathogenic mechanisms, such as alteration of autophagy, increased senescence, telomere shortening, or genomic instability, all of them hallmarks of aging [9], have been proposed to explain how the expansion in the CTG repeat of affected patients leads to the DM multisystem phenotypes [7]. However, detailed experimental validation of these mechanisms remains incomplete and has not yet been clarified.

It is well known the existence of several metabolic alterations, which accumulate over time, that affect longevity, aging and neurodegeneration [10, 11]. As a consequence, deregulated nutrient sensing and mitochondrial dysfunction have been proposed as hallmarks of aging [9] and metabolism is a pillar of aging [12]. In DM1, patients present several metabolic defects such as hyperinsulinemia, glucose resistance, and, in some cases, diabetes mellitus [7]. Moreover, muscle samples *in vitro* and blood samples *in vivo* show reduced Coenzyme Q10 (CoQ10) levels, a component of the electron transport chain that participates in aerobic cellular respiration [13, 14], which is indicative of mitochondrial dysfunction. However, the role of metabolism and mitochondria in the pathogenesis of DM1 has not been addressed in detail. In this work, we studied their contribution using human primary fibroblasts and peripheral blood mononuclear cells (PBMCs) derived from healthy donors and patients with DM1 as models. Our results indicated that DM1 fibroblasts showed impaired metabolism and mitochondrial dysfunction resulting in lower levels of ATP production and increased reactive oxygen species (ROS) production. PBMCs from DM1 patients also showed impaired mitochondrial dynamics and energy homeostasis. Interestingly, treatment with metformin resulted in the restoration of these phenotypes.

## RESULTS

### DM1-derived fibroblasts present impaired metabolism

**Figure 1:**
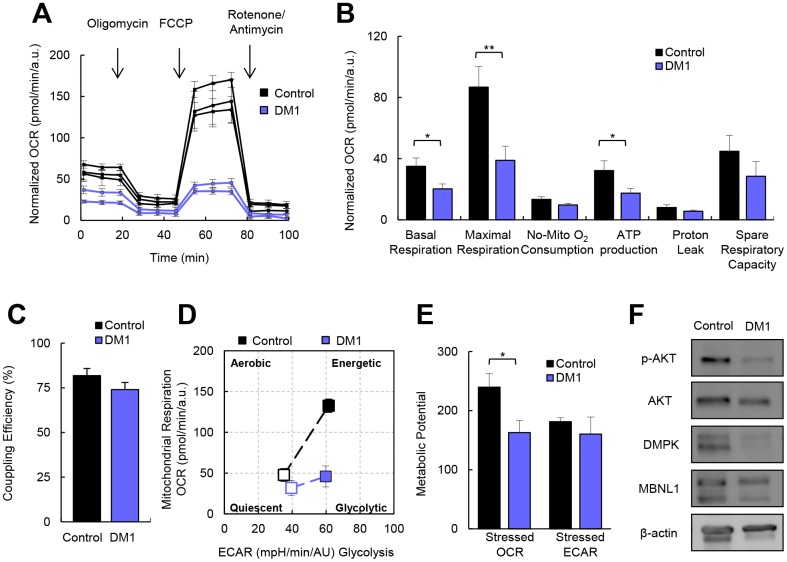
**DM1-derived fibroblasts present impaired metabolism.** (**A**) Kinetic normalized OCR response in DM1 and control fibroblasts in basal conditions and after consecutive addition of Oligomycin 1.5 μM, FCCP 1.5 μM and Antimycin-A/Rotenone 1.5 μM. A representative experiment out of 3 is shown with 3 independent control cultures and 2 DM1. (**B**, **C**) Quantification of mitochondrial respiratory functions and coupling efficiency in DM1 (n=7) and control fibroblasts (n=3). (**D**) Representative energy map and (**E**) Quantification of metabolic potential of DM1 and control fibroblasts. *Stressed* indicates the values of OCR and ECAR after the injection of oligomycin and FCCP simultaneously. Results are obtained from controls (n=3) and DM1 (n=5) cultures. (**F**) Representative immunoblots of phospho-AKT, AKT, DMPK and MBNL1 in DM1-derived fibroblasts and healthy controls (n=3).

To investigate the role of cellular metabolism in the pathogenesis of DM1, we first measured the oxygen consumption rate (OCR) in the fibroblasts of patients with DM1 and healthy donors. DM1 fibroblasts showed a 40% and 50% reduction in basal respiration and maximal respiration, respectively, compared to controls, which leads to a 50% reduction in ATP production via the Mitochondrial Oxidative Phosphorylation System (OXPHOS) activity (Figure 1A, 1B). Next, we hypothesized that the reduction in OXPHOS activity could be responsible for a reduction in the glycolysis pathway. To examine this hypothesis, we measured extracellular acidification (ECAR) as a measure of glycolysis [15]. We did not find any alteration in the glycolysis pathway (Supplementary Figure 1A, 1B), suggesting that all glucose taken by DM1 fibroblasts was coupled to pyruvate production.

The addition of carbonyl cyanide-4 (trifluoromethoxy) phenylhydrazone (FCCP) simulates an exacerbated physiological energy demand by stimulating the respiratory chain to operate at maximum capacity. DM1 cells were not able to respond to this stress as efficiently as controls, farther indicating impaired maximal respiration (Figure 1A, 1B). However, we did not find any difference in the proton-leak nor the coupling efficiency (Figure 1A–1C). Therefore, it seems that all the protons generated are coupled to ATP production. Moreover, DM1 fibroblasts have a more quiescent metabolism compared to healthy controls. In addition, after simulating a stress, DM1 fibroblasts could not switch to a more energetic metabolism (Figure 1D, 1E), resulting in a lower metabolic potential. Consistent with these results, DM1 fibroblasts presented lower AKT activation (measured as phosphorylated AKT) (Figure 1F), which is the central mediator of the PI3K pathway that serves a key role in multiple cellular processes, including glucose metabolism [16]. In summary, DM1-derived fibroblasts present decreased cellular metabolism.

### Correlation between impaired metabolism and markers of disease pathophysiology

**Figure 2:**
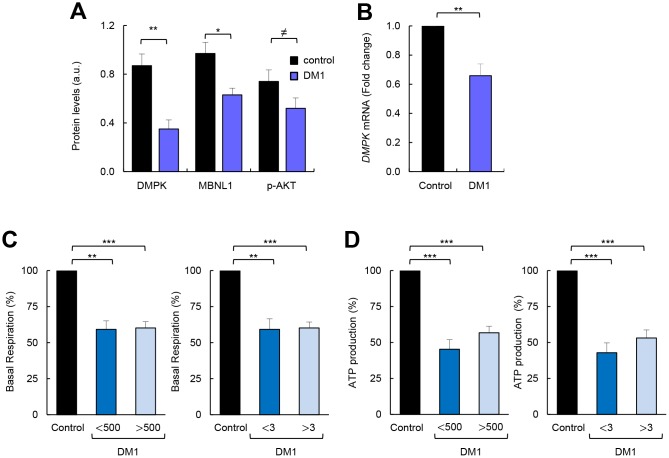
**Correlation between impaired metabolism and markers of disease pathophysiology.** (**A**) Quantification of protein levels shown in Figure 1F (n=4). (**B**) mRNA levels of *DMPK* in DM1 fibroblasts (n=7) and controls (n=3). (**C**) Basal respiration levels in controls (n=3) and DM1 fibroblasts stratified by CTG expansion in <500 (n=4) and >500 (n=3) (left) and MIRS scale in <3 (n=2) and >3 n=5 (right). (**D**) ATP production levels using the same stratification.

Next, we attempted to associate the impaired metabolism of DM1-derived fibroblasts with several pathophysiological characteristics of the disease. First, we found that the decrease in AKT phosphorylation in DM1-derived fibroblasts correlated with lower expression of DMPK and MBNL1, both at protein (Figure 2A) and mRNA (Figure 2B) levels . Moreover, we examined whether there was a correlation between the severity of the metabolic alterations and both the number of CTG expansions and the Muscular Impairment Rating Scale (MIRS) score. We did not detect significant differences in basal and maximal respiration or in ATP production when fibroblasts were divided into those with less or more than 500 CTG repeats and 3 MIRS score (Figure 2C, 2D, Supplementary Figure 2). Moreover, there were no marked differences between cells obtained from DM1 patients of different ages, although the cells from a 71 year-old patient showed slightly higher impairment than others (Table 1, Supplementary Figure 3). Overall, metabolic dysfunction in fibroblasts derived from patients with DM1 seems not to be significantly altered by the repeat expansion of these patients.

**Table 1:** Characteristics of human primary fibroblasts.

**Fibroblasts**	**Status**	**Gender**	**Age biopsy (years)**	**MIRS**	**CTG (n) in blood**	**Age diagnosis (years)**
C1	Control	M	49			
C2	Control	F	48			
C3	Control	Unknown	53			
DM1-1	DM1 patient	M	71	3	167	53
DM1-2	DM1 patient	F	45	2	333	41
DM1-3	DM1 patient	F	59	3	333	26
DM1-4	DM1 patient	F	44	2	833	27
DM1-5	DM1 patient	M	56	3	1333	20
DM1-6	DM1 patient	F	34	4	1650	12
DM1-7	DM1 patient	M	50	5	233	20

MIRS: Muscle impairment rating scale; M: male; F:female.

### DM1-derived fibroblasts display mitochondrial dysfunction but no changes in mitochondrial biogenesis

**Figure 3:**
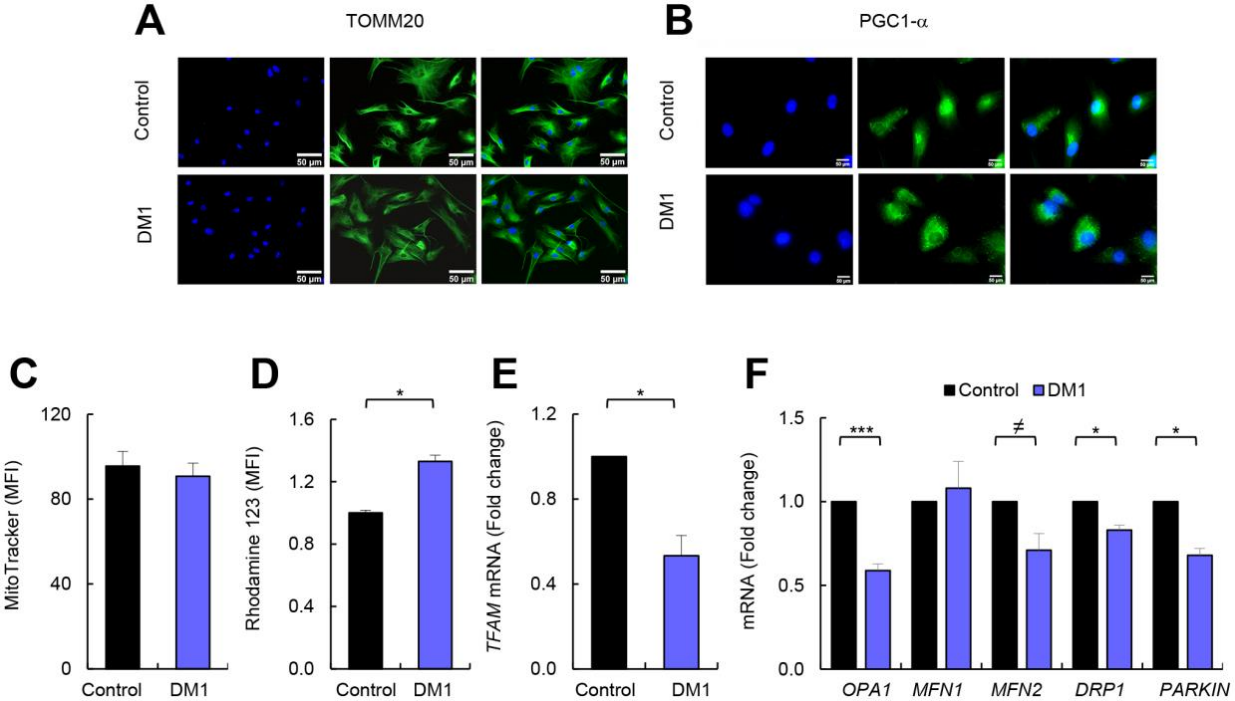
**DM1-derived fibroblasts have no changes in mitochondria biogenesis.** (**A**, **B**) Representative images of immunofluorescence of TOMM20, and PGC1-α in DM1 and control fibroblasts (n=3). (**C**) Medium fluorescence intensity of MitoTracker Red FM in control (n=3) and DM1 cells (n=5) and (**D**) of Rhodamine 123 in DM1 and control fibroblasts (n=3). (**E**) mRNA levels of *TFAM* transcription factor (n=3). (**F**) mRNA levels of *OPA1*, *MFN1*, *MFN2*, *DRP1* and *PARKIN* in DM1 and control fibroblasts (n≥2).

The results presented above indicate that the mitochondria of patients with DM1 could function normally, but with a reduced OXPHOS activity. We further investigated this by examining the biogenesis of mitochondria [17]. First, we evaluated the levels of two markers of mitochondrial content and biogenesis such as TOMM20 and PGC1-α. Immunofluorescence showed that expression of these two markers was not markedly altered in cells from DM1 compared to healthy controls (Figure 3A, 3B). In addition, flow cytometry was used to analyze another marker of mitochondrial content, MitoTracker, obtaining similar results. Indeed, no differences were detected in the mitochondrial content in DM1 and control cells (Figure 3C). Moreover, the mitochondrial membrane potential remained elevated in DM1 cells (Figure 3D). However, the expression of the mitochondrial transcription factor A (*TFAM*) gene that participates in the regulation of the mitochondrial genome [18], was reduced by 50% in DM1 fibroblasts (Figure 3E). These results suggest that the impaired cellular bioenergetics were not related to substantial alterations in mitochondrial biogenesis and content.

Mitochondria are organelles with high dynamic plasticity to rapidly adapt in response to stress situations. Mitochondrial dynamic is regulated by a machinery of pro-fusion and -fission proteins, which constitutes an important part of the mitochondria quality control as it facilitates the elimination of damaged mitochondria by mitochondrial selective autophagy (mitophagy) [19]. We studied the expression of *OPA1*, *MFN1* and *MFN2* fusion related genes, *DRP1* fission related gene, and *PARKIN*, which is involved in mitophagy [20]. Interestingly, the levels of *OPA1*, *MFN2*, *DRP1,* and *PARKIN* were decreased in DM1-derived fibroblasts (Figure 3F). Overall, DM1-derived fibroblasts show mitochondrial dysfunction.

### DM1-derived blood samples show mitochondria dysfunction

**Figure 4:**
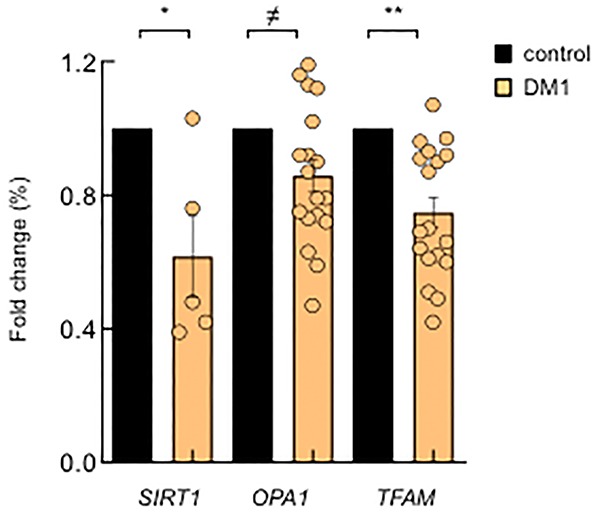
**DM1-derived blood samples also show mitochondria dysfunction.** mRNA levels of *SIRT1*, *OPA1* and *TFAM* in PBMCs derived from DM1 (n≥12) and controls (n=4).

Next, we investigated whether these results could be translated to the clinical setting. Therefore, we measured the expression levels of several of the aforementioned genes in PBMCs from a cohort of patients with DM1 established in Guipuzcoa (Basque Country, Spain) [21]. Interestingly, we found lower expression levels of *SIRT1*, a key metabolic sensor that modulates a large variety of cellular processes such as energy metabolism stress response and aging [22], *OPA1* and *TFAM* (Figure 4), further supporting the relevance of the results obtained in cell culture and highlighting the importance of metabolism and mitochondria for the disease.

### DM1-derived fibroblasts present accumulation of ROS and p38MAPK activation

**Figure 5:**
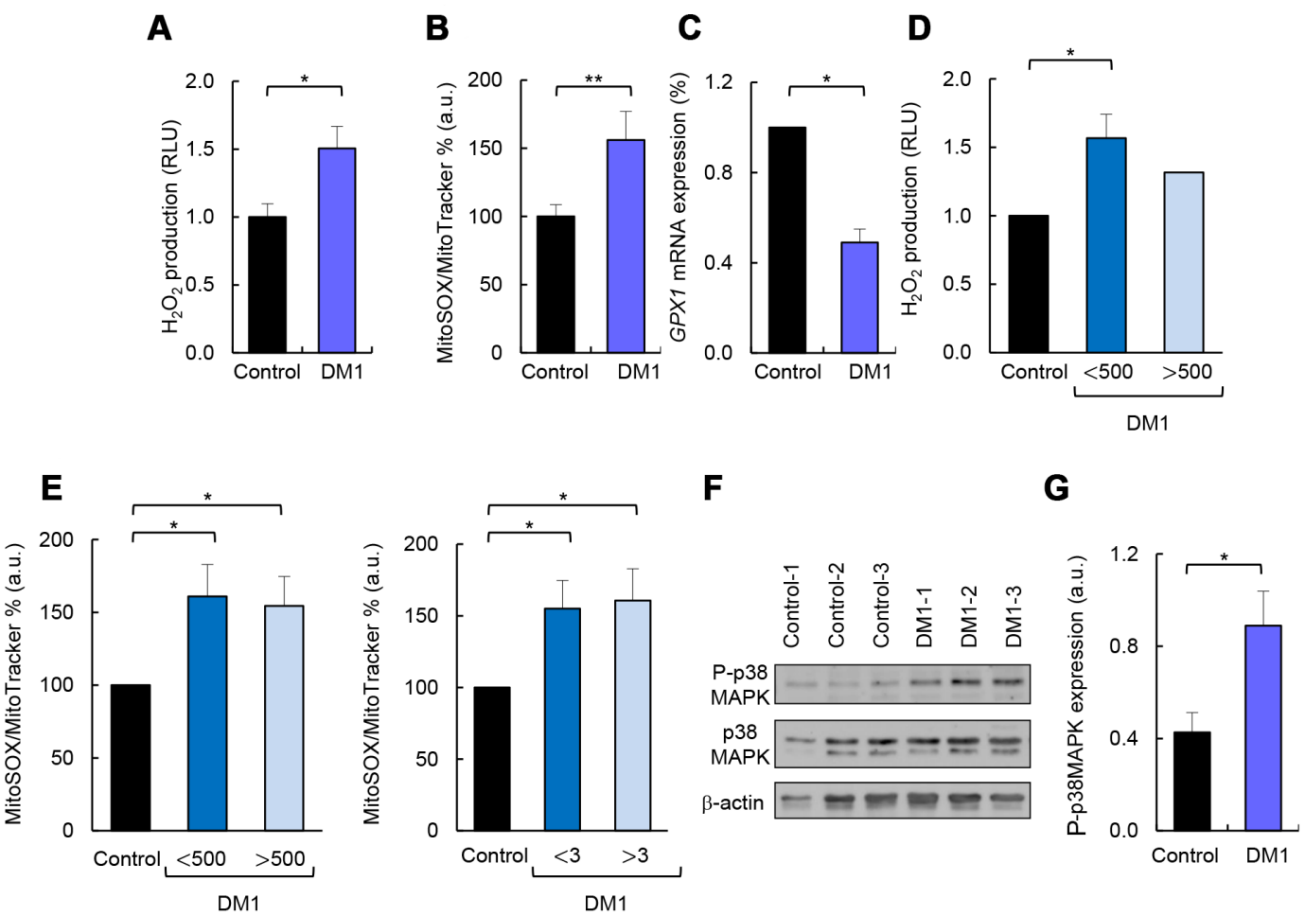
**DM1-derived fibroblasts present accumulation of ROS and p38MAPK activation.** (**A**) Luminescence signal proportional to H_2_O_2_ production in DM1 (n=4) and control fibroblasts (n=3). (**B**) Medium fluorescence intensity of MitoSOX+ values normalized to mean fluorescence of MitoTracker values in controls (n=3) and DM1 (n=5). (**C**) *GPX1* mRNA levels in DM1 and control fibroblasts (n≥2). (**D**) Luminescence signal proportional to H_2_O_2_ production in controls (n=3) and DM1 fibroblasts stratified by CTG expansion in <500 CTG (n=3) and >500 CTG (n=1). (**E**) Medium fluorescence intensity of MitoTracker Red FM in controls (n=3) and DM1 stratified by CTG expansion in <500 (n=3) and >500 (n=2) (left) and MIRS scale in <3 (n=2) and >3 (n=3) (right). (**F**, **G**) Representative immunoblot and quantification of P-p38MAPK and p38MAPK protein levels in DM1 and control fibroblasts (n=3).

Production of ROS is enhanced in several pathological conditions in which the respiratory chain is impaired [23]. Therefore, we measured ROS production and found a 50% increase in total ROS in DM1 cells compared to controls (Figure 5A). Similar results were obtained when specific ROS produced by the mitochondria were measured in DM1 fibroblasts and compared to controls (Figure 5B). Further, the expression of glutathione peroxidase 1 (*GPX1*) antioxidant gene was decreased by 50% in DM1 cells (Figure 5C). In agreement with metabolic studies, we did not observe differences in ROS accumulation when fibroblasts were divided based on the number of CTG repeats and MIRS score (Figure 5D, 5E).

p38MAPK is a stress-activated protein kinase, which accumulates with aging and it is activated by the presence of ROS [24, 25]. Consequently, we measured the total levels of p38MAPK and its phosphorylated form (P-p38MAPK) and found an over 2-fold increase in the levels of P-p38MAPK in DM1-derived fibroblasts compared to control cells (Figure 5F, 5G). In summary, DM1 fibroblasts display increased ROS production, which is associated with an enhanced activation of p38MAPK.

### Metformin restores metabolism and mitochondria activity

**Figure 6:**
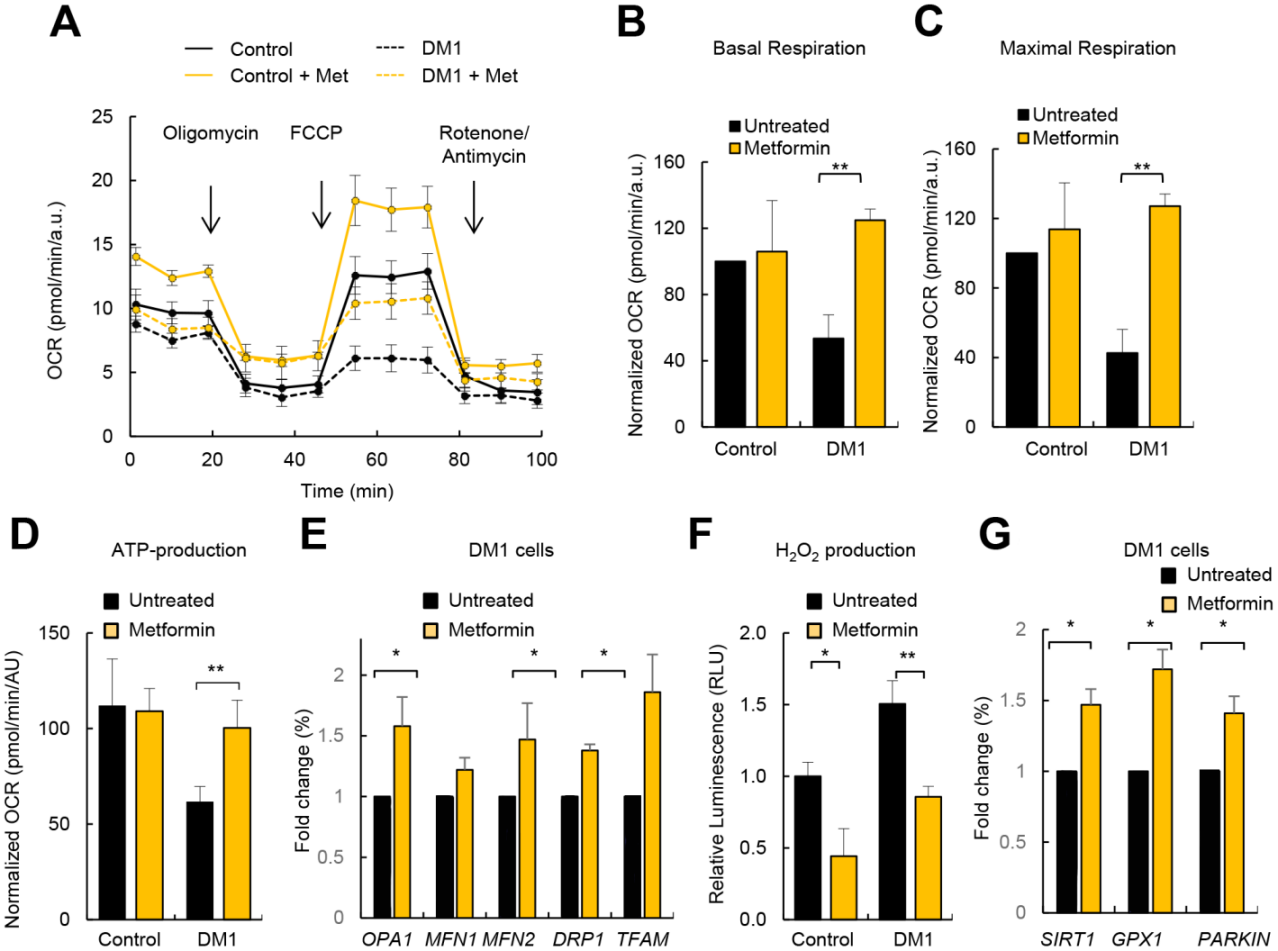
**Metformin restores OXPHOS activity and ROS production in DM1 fibroblasts.** (**A**) Representative kinetic normalized OCR response in DM1 (n=6) and control fibroblasts (n=3) after treatment with 1 μM of metformin for 72 h. DM1 and control fibroblasts were plated at 5.000 cells/well 24 hours prior to the assay. A representative experiment out of 3 is shown. (**B**–**D**) Quantification of mitochondrial basal respiration, maximal respiration, and ATP production respectively after treatment with 1 μM of metformin for 72 h of controls (n=3) and DM1 fibroblasts (n=6). (**E**) mRNA levels of *OPA1*, *MFN1*, *MFN2*, *DRP1* and *TFAM* after treatment with 1 μM of metformin for 72 h (n≥2). (**F**) H_2_O_2_ production after treatment with 1 μM of metformin for 72 h (n=3). (**G**) mRNA levels of *SIRT1*, *GPX1* and *PARKIN* in DM1 and control fibroblasts after treatment with 1 μM of metformin 72 h (n≥2).

Metformin is a first-line anti-diabetic agent that functions mainly through the suppression of glucose production and alleviation of insulin resistance and has recently been shown to improve mitochondrial respiratory activity [26, 27]. We examined whether metformin could improve the impaired OXPHOS activity in patients with DM1. To test this idea, we treated DM1 and control fibroblasts with 1 μM of metformin for 72 hours and evaluated cellular metabolism and mitochondrial activity. Interestingly, metformin improved the basal oxygen consumption rate and maximal respiration of DM1 fibroblasts by more than twice (Figure 6A–6C), which resulted in an increased ATP production via OXPHOS (Figure 6A, 6D). Moreover, it increased the levels of *OPA1*, *MFN2*, *DRP1* and *TFAM* in DM1 cells by at least 1.5-fold (Figure 6E).

Next, we measured ROS production and found that treatment with metformin significantly decreased ROS production in control as well as in DM1 fibroblasts (Figure 6F). In accordance, the levels of *GPX1* and *PARKIN* were elevated in the presence of metformin in DM1 cells (Figure 6G). The levels of *SIRT1*, a critical downstream target, were also induced in DM1 cells cultured in the presence of metformin validating the effect of metformin in metabolic pathways (Figure 6G). In summary, metformin restores the impaired metabolism and mitochondrial activity in DM1 fibroblasts.

### Metformin restores additional DM1-associated phenotypes

**Figure 7:**
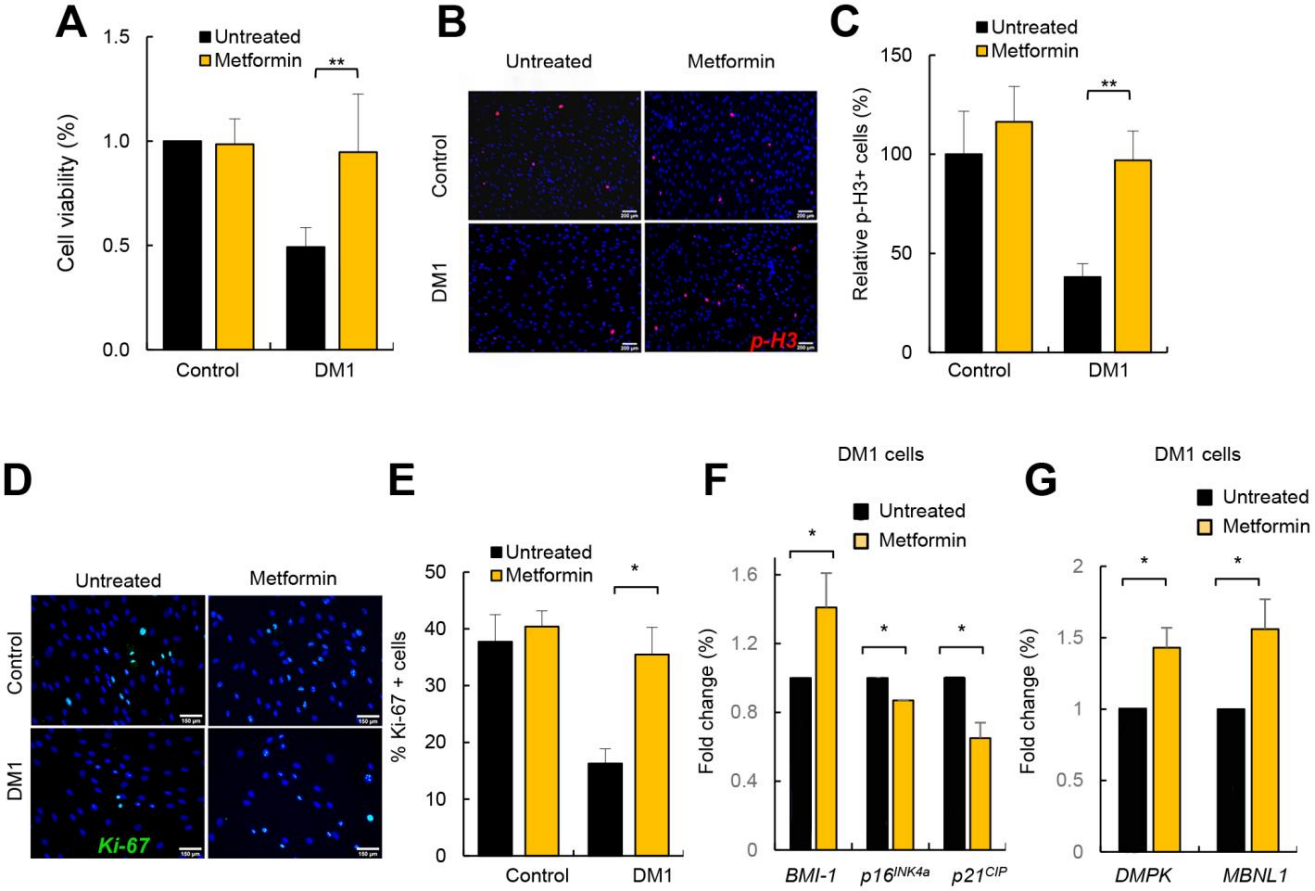
**Metformin restores cell viability and proliferation in DM1 fibroblasts.** (**A**) Cell viability of DM1 (n=5) and control (n=3) fibroblasts measured in MTT studies after treatment with 1 μM of metformin for 72 h. (**B**, **C**) Representative image and quantification of p-H3 (Ser10) staining in the same conditions in controls (n=3) and DM1 (n=7). (**D**, **E**) Representative image of Ki-67 staining and quantification in controls (n=2) and DM1 cells (n=3). (**F**) mRNA levels of *BMI-1*, *p16^INK4a^* and *p21^CIP^* in cells treated or not with 1 μM of metformin for 72 h (n≥2). (**G**) mRNA levels of *DMPK* and *MBNL1* in the same conditions (n=3).

Metformin exerts a potent anti-aging activity, in part by increasing proliferation and inhibiting senescence [28, 29]. It has been previously reported that DM1 fibroblasts display decreased cell proliferation and enhanced senescence accumulation [8, 30]. Next, we investigated the impact of metformin in the proliferative potential of DM1 fibroblasts. For this, we treated DM1 and control fibroblasts with 1 and 10 μM of metformin and measured cell viability. As expected, DM1 cells had lower viability than controls but, importantly, the treatment increased significantly the viability of DM1 fibroblasts, reaching almost the levels of control cells (Figure 7A and Supplementary Figure 4). Moreover, we measured the number of cells positive for phospho-Histone H3 (p-H3) and Ki-67, which are well-established markers of mitosis and cell division, respectively, and found reduced numbers in both markers in DM1 cells (Figure 7B–7E). Importantly, metformin increased the number of p-H3 and Ki-67 positive cells by almost 3-fold in DM1 cells (Figure 7C, 7E). These functional results were further validated at the molecular level. Metformin modulated the expression of critical genes involved in cell proliferation and cell cycle activity such as *BMI-1, p16^INK4a^* and *p21^CIP^*. In particular, treatment for 72 h increased the levels of *BMI-1,* and partially decreased the levels of *p16^INK4a^* and *p21^CIP^* cell cycle inhibitors (Figure 7F). Finally, we also detected that metformin restored by 1.5-fold the levels of *DMPK* and *MBNL1* (Figure 7G). Thus, metformin rescues multiple phenotypes associated to DM1 cells.

## DISCUSSION

We established primary cultures of fibroblasts derived from patients with DM1 and found that they display impaired metabolism and mitochondrial dysfunction. In particular, DM1 fibroblasts present lower production of ATP by OXPHOS, less efficient mitochondrial electron transport chain, impaired mitochondrial dynamics, and higher production of ROS compared with healthy control-derived fibroblasts. Interestingly, some of these defects, such as energy homeostasis and mitochondrial dynamics, were also detected in PBMCs from patients with DM1, together revealing the impact of metabolism and mitochondrial function on the pathophysiology of DM1.

These results show that fibroblasts, which are a well-established model for cell aging studies *in vitro* [31], might be a good cellular model to characterize the pathophysiology of the disease, as they resemble multiple molecular and cellular phenotypes of the disease. However, we did not detect a correlation between the severity of the phenotypes and the number of CTG repeats. This result might be potentially biased by methodological reasons because CTG expansion was measured several years before isolation of skin fibroblasts and blood samples. In addition, some experiments were performed at early passage.

Our results reveal novel processes involved in the pathophysiology of the disease. Indeed, the role of mitochondria in DM1 remained practically unknown. A previous study observed an inverse correlation between the expression of CoQ10, an electron carrier in the mitochondrial respiratory chain, and lactate production with CTG expansion in PBMC samples [14], whereas mitochondrial dysfunction was suggested to occur in muscles of patients with DM1 as well [32]. The results of these studies are in line with our data and are indicative of mitochondrial dysfunction in DM1. The lower mitochondrial efficiency detected in our study could be due to the conversion of pyruvate, generated during glycolysis, to lactate instead of acetyl CoA, which is transported to mitochondria and enters into the Krebs cycle. Moreover, our results show that mitochondrial biogenesis seems to be normal in DM1 cells, but they are not able to maintain the metabolic state as a consequence of unbalance remodeling of mitochondrial network morphology, which is not correctly controlled by the machinery of pro-fusion and fission proteins, and impaired elimination through mitophagy.

DM1 patients develop a large variety of symptoms in multiple systems that strongly resemble the clinical signs of accelerated aging, including some related to metabolism and mitochondria dysfunction such as insulin resistance, glucose intolerance, hyperinsulinemia, and increased risk of type 2 diabetes [33, 34]. Our results shed light in the underlying molecular mechanisms of these symptoms. Given that mitochondria is the main energy hub of the cell and the main intracellular source of ROS, our results might be extended to additional DM1 symptoms, particularly in the muscle, since a shift in energy production anticipates muscle atrophy with aging. Finally, our results further support the link between DM1 and accelerated aging [8], since cellular metabolism and mitochondrial dysfunction are critical mechanisms in aging.

DM1 is a rare, clinically variable disease with no currently available treatment to slow or stop disease progression. Supportive treatments, preventive measures and clinical surveillance are the only options available for patients with DM1 [35]. Metformin is a synthetic biguanide that is currently one of the most recommended medications for type 2 diabetes treatment around the world. Interestingly, studies in both vertebrates and invertebrates have shown that metformin delays aging and increases longevity [29]. Moreover, a meta-analysis has suggested that metformin reduces all-cause mortality and aging-related diseases in humans independent of its effect on diabetes [36]. We show here that metformin improves ATP production by OXPHOS and decreases the production of ROS in DM1 cells even at a much lower concentration compared to its current therapeutic dose (1 μM *vs* 75 μM). In line with our results, it has been recently shown that mitochondria might be a target of metformin [27]. We also found that metformin treatment reverses additional DM1-related phenotypes such as impaired proliferation, suggesting that its mechanism of action in DM1 is wider. In support, low doses of metformin may also correct several alternative splicing defects in DM1 myoblasts *in vitro* [37], the use of metformin reduced the risk of cancer in patients with DM1 having diabetes [38], and also improved mobility of DM1 patients in a small randomized clinical trial [39]. If the hypothesis of an accelerated aging in patients with DM1 is validated, our results could be added to the potential benefits of expanding metformin use in DM1, outside of the management of T2D, to include cancer prevention [38] and also phenotypes associated with aging. In summary, our results showed the efficacy of metformin in a pre-clinical setting and suggest that it warrants further assessment as a candidate drug for DM1 treatment.

## MATERIALS AND METHODS

### Study approval

This study was approved by the Donostia University Hospital Ethical Board (approval number 15-57) and was conducted in accordance with the Declaration of Helsinki’s ethical standards. All subjects gave written informed consent before sample donation.

### Reagents and cell culture

For the isolation of primary fibroblasts, punch skin biopsies were chopped into 2–3 mm^3^ fragments and placed on a surface moistened with modified Eagle’s medium containing 13% newborn calf serum, 0.4% penicillin/streptomycin (Gibco, Waltham, MA, USA) and 2 mM L-glutamine (Gibco). Flasks were incubated vertically for 3–6 hours at 37 °C in a 5% CO_2_ atmosphere and then returned to the horizontal position. Human fibroblasts were cultured in Dulbecco’s Modified Eagle Medium (DMEM, Gibco) containing 10% fetal bovine serum (FBS) (Sigma-Aldrich, St Louis, MO, USA), 1% L-glutamine (Gibco) and 1% penicillin/streptomycin (Gibco). 7 independent cultures from different patients with DM1 and 3 from healthy controls were established (see Table 1 for patient characteristics). When indicated, fibroblasts were treated with metformin (Sigma-Aldrich) for 72 hours. Experiments were performed in early passage cultures (range of 5 to 10 passages).

### Metabolic measurements

Measurement of OCR and ECAR were performed in XF96 plates with XF Extracellular Flux Analyzer (Seahorse Bioscience). Fibroblasts were seeded in collagen (BD Biosciences) coated XF 96-well plates (Seahorse/Agilent) in octuplicates at 1.2x10^4^ cells/well in 100 μl of growth medium. Mitochondrial activity was evaluated using the *Seahorse XF Cell Mito stress Test Kit,* according to manufacturer’s instructions (Agilent). In the metformin treatment experiments, cells were plated at 5x10^3^ cells/well 24–28 hours prior to the assay. Oligomycin (75351, Sigma-Aldrich), FCCP (C2920, Sigma-Aldrich), and Rotenone/Antimycin A (R8875 and A8674, Sigma-Aldrich) were used at 1.5 μM concentration, after a titration experiment. Glycolytic activity was evaluated using the *XF Glycolysis Stress Test* according to manufacturer’s instructions (Agilent). Glucose (G8769, Sigma-Aldrich) was used at 10 mM, oligomycin at 1 μM and 2-D-Deoxy-Glucose at 50 mM (D6134, Sigma-Aldrich). Cell content was normalized using crystal violet. The post-normalization values of OCR and ECAR reflect both the metabolic activities of the cells and the number of cells being measured. Data were further processed according to manufacturer’s instructions.

### Total ROS measurement

A total of 1x10^3^ fibroblasts were plated in 96-well plates and grown for 3 days. Afterwards, *ROS-Glo H_2_O_2_* *Assay* (G8820, Promega) was performed according to the manufacturer’s instructions. Briefly, a H_2_O_2_ substrate reacts directly with H_2_O_2_ to generate a luciferin precursor and, upon addition of a detection reagent, this precursor is converted to luciferin, which generates a luminescent signal that is proportional to the H_2_O_2_ concentration. White flat bottom plates (Corning) were used for final readout in a PHERAstar (BMG Labtech) luminometer plate reader.

### Mitochondrial ROS production and mitochondrial content measurement

Mitochondrial ROS analysis was performed using the dye *MitoSOX* (M36008, Invitrogen). Mitochondrial content was assayed using the dye *MitoTracker FM* (M22425, Invitrogen), which passively diffuses across the plasma membrane and accumulates in active mitochondria.

20x10^4^ fibroblasts per condition were grown for two days, reaching 70% confluence in p100 plates. Cells were detached using trypsin for 5 min at 37 °C. For MitoSOX staining, cells were washed once using warm HBSS, incubated with 5 μM of MitoSOX in HBSS for 30 min at 37 °C, washed 3x using warm HBSS and suspended in HBSS. For MitoTracker staining, cells were washed with PBS, incubated with 0.2 μM MitoTracker for 30 min at 37 °C, washed 3x using warm PBS and suspended in PBS. Cells were directly analyzed via flow cytometry. In FSC and SSC, we first gated the population; next, two gates were set on SSC-A vs. SSC-H and SSC-A vs. SSC-W to exclude doublets. Based on an unstained control, MitoSOX+ and MitoTracker+ gates were set. Mean fluorescence of MitoSOX+ was normalized as a mean fluorescence of MitoTracker values, which represents ROS production per mitochondria. Antimycin was used as a positive control and FCCP as a negative control.

### Mitochondrial membrane potential measurement

20x10^4^ fibroblasts per condition were grown for 2 days, reaching 70% confluence in p100 plates. Cells were detached using trypsin for 5 min at 37 °C. We used 1 μM of *Rhodamine 123* (Invitrogen) for 15 min at 37 °C to measure the mitochondrial membrane potential. This probe is readily sequestered by functioning mitochondria and is easily washed out of cells once the mitochondria experience a loss in membrane potential.

### Cell viability

Fibroblasts were seeded in 96-well plates followed by treatment with metformin for 72 h. Viable cells were quantified using the modified 3-(4,5-dimethylthiazol-2-yl)-2,5-diphenyltetrazolium bromide (MTT) (Sigma-Aldrich) assay in six replicates per condition.

### mRNA expression analysis

Total RNA was extracted using TRIzol (Life Technologies). Reverse transcription was performed using random priming and the *Maxima First Strand cDNA Synthesis Kit* for RT-qPCR, with dsDNase (Thermo Fisher Scientific, Waltham, MA, USA), according to the manufacturer’s guidelines. Quantitative PCR was performed using Power SYBR Green PCR Master Mix (Thermo Fisher Scientific), 10 mM of each primer and 20 ng of cDNA, in a CFX384 thermocycler (Bio-Rad, Hercules, CA, USA). Primer sequences will be given upon request. Variations in RNA input were corrected by analyzing the expression of *GAPDH* as a housekeeping gene. The ΔΔCT method was used for relative quantification.

### Western blot and immunofluorescence analysis

Immunoblot and immunofluorescence assays were performed following standard procedures, as previously described [40]. Primary antibodies were: phospho Histone H3 Ser10 (ab14955, Abcam), TOMM20 (11802-1-AP, Proteintech), PGC1-α (NBP1-04676, Novus Biologicals), phospho p38MAPK Thr180/Tyr182 (9211, Cell Signaling), p38MAPK (sc-7972, Santa Cruz Biotechnology) AKT1/2/3 (sc-8312, Santa Cruz Biotechnology), phospho AKT Ser 473 (9271, Cell Signaling), DMPK (sc-134319, Santa Cruz Biotechnology), MBNL1 (ab45899, Abcam) and β-actin (AC-15, Sigma-Aldrich). For western blot detection of primary antibodies, we used HRP-linked antibodies (Santa Cruz Biotechnology) and the detection was performed by chemiluminescence using *Novex ECL Chemi Substrate* (Thermo Fisher). For immunofluorescence, nuclear DNA was stained with Hoechst (33342, Sigma-Aldrich).

### Statistics

Data are presented as mean values ± S.E.M., with the number of experiments (n) in parentheses. Unless otherwise indicated, statistical significance (p-values) was calculated using the Student´s t -test. Asterisks (*, **, and ***) indicate statistical significance (p < 0.05, p < 0.01, and p < 0.001, respectively).

## Supplementary Material

Supplementary Figures
