# Supplementary material for: Myotonic Dystrophy type 1 cells display impaired metabolism and mitochondrial dysfunction that are reversed by metformin
Source: Aging (Albany NY). 2020 Apr 8;12(7):6260–75. doi: 10.18632/aging.103022 (PMC7185118; doi:10.18632/aging.103022)
Supplement: Supplementary Figures [file aging-12-103022-s001..pdf]

SUPPLEMENTARY FIGURES

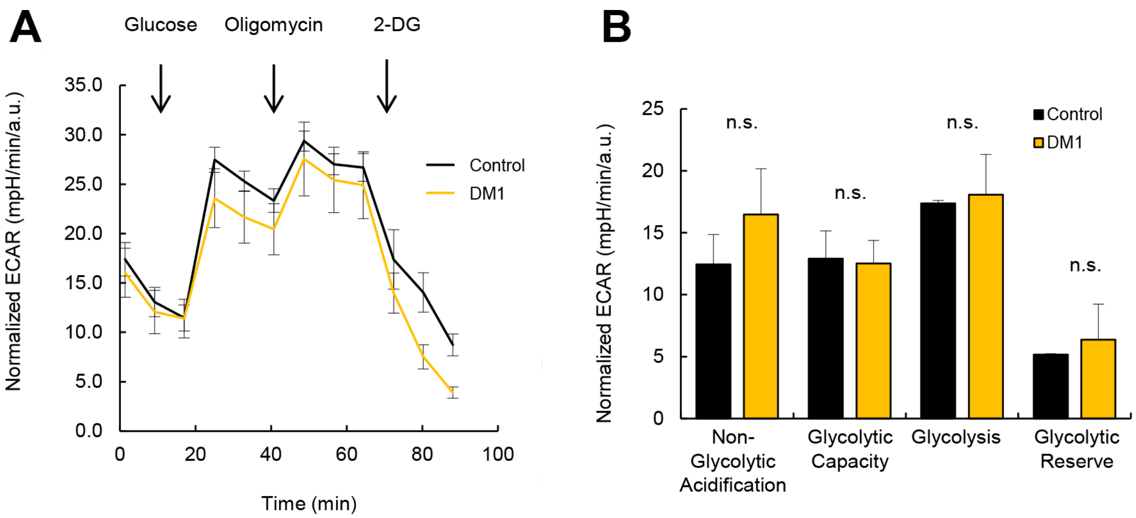

Supplementary Figure 1. DM1-derived fibroblasts have no changes in glycolysis. (**A**) Kinetic normalized ECAR response in DM1 and control fibroblasts obtained in basal conditions and after consecutive addition of Glucose 10 mM, Oligomycin 1 µM and 2-D-Deoxy-Glucose 50 mM. DM1 and control fibroblasts were plated at 5x10^3 cells/well, in XF96 cell culture plates, 24–28 h prior to the assays. The assay medium was the substrate-free base medium supplemented with 2 mM glutamine. Upon completion of an assay, cells were normalized using violet crystal. A representative experiment is shown here (n=3 controls and n=4 DM1 patients). (**B**) Quantification of glycolytic functions in DM1 and control fibroblasts (n=3 controls and n=4 DM1 patients).

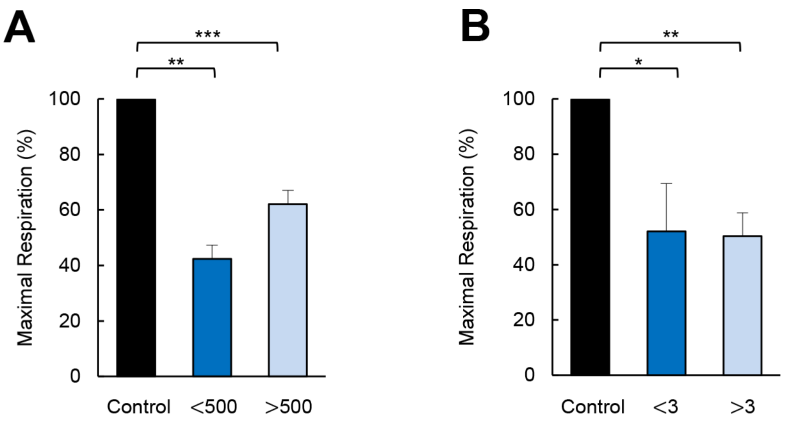

Supplementary Figure 2. No differences in maximal respiration of DM1 fibroblasts stratified by CTG amplification and MIRS scale. (**A**) Maximal respiration in fibroblasts derived from controls (n=3) and DM1 patients stratified by CTG expansion in <500 CTG (n=4) and >500 (n=3). (**B**) Maximal respiration in fibroblasts derived from controls (n=3) and DM1 patients stratified by MIRS scale in <3 (n=2) and >3 (n=5).

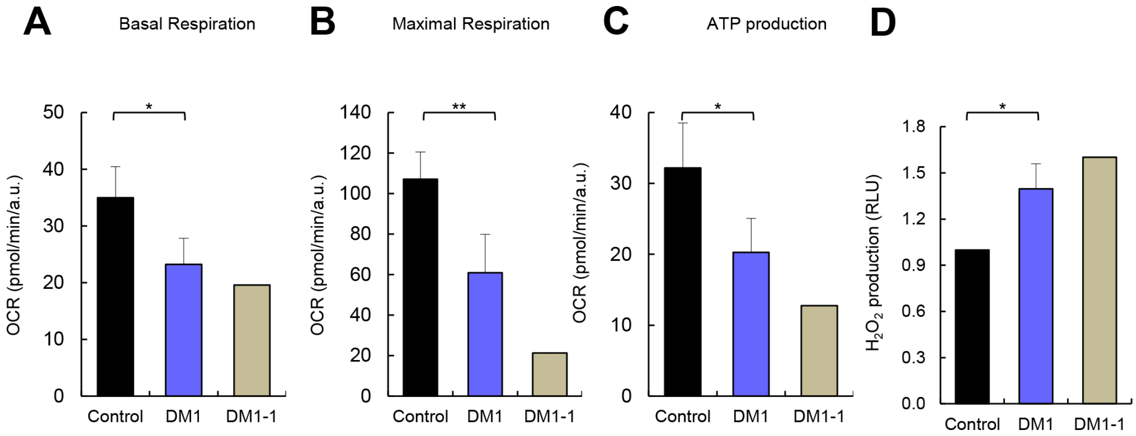

Supplementary Figure 3. Comparison of results in DM1 fibroblasts based on patient age. (**A**) Analysis of Basal respiration (**B**) Maximal respiration, (**C**) ATP production and (**D**) H_2O_2 production in control fibroblasts (control; black) (n=3), in DM1-derived fibroblasts excluding a 71 years-old case (DM1; blue) (n=6), and cells derived from a 71 years-old patient (DM1; grey).

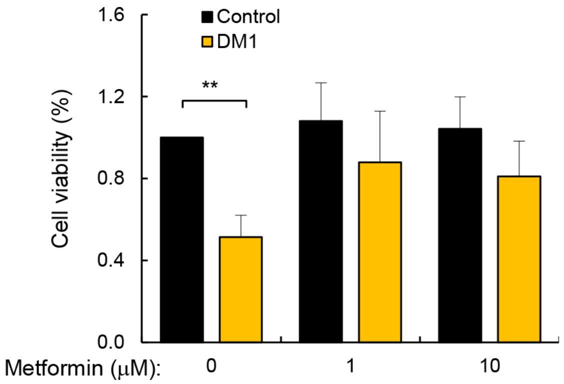

Supplementary Figure 4. Metformin restores cell viability in DM1-derived fibroblasts. Cell viability after treatment with 1 and 10 mM of metformin for 72 h. Figure shows results from controls (n=3) and DM1 (n=5) cells.
